# Missense mutations in spike protein of SARS‐CoV‐2 delta variant contribute to the alteration in viral structure and interaction with hACE2 receptor

**DOI:** 10.1002/iid3.683

**Published:** 2022-08-17

**Authors:** Tousif Bin Mahmood, Mohammad Imran Hossan, Shafi Mahmud, Mst. Sharmin Sultana Shimu, Md. Jahidul Alam, Md. Mahfuzur Rahman Bhuyan, Talha Bin Emran

**Affiliations:** ^1^ Department of Biotechnology and Genetic Engineering Noakhali Science and Technology University Noakhali Bangladesh; ^2^ Department of Genetic Engineering and Biotechnology University of Rajshahi Rajshahi Bangladesh; ^3^ Department of Applied Chemistry and Chemical Engineering Noakhali Science and Technology University Noakhali Bangladesh; ^4^ Department of Biochemistry and Molecular Biology Noakhali Science and Technology University Noakhali Bangladesh; ^5^ Department of Pharmacy BGC Trust University Bangladesh Chittagong Bangladesh; ^6^ Department of Pharmacy Faculty of Allied Health Sciences, Daffodil International University Dhaka Bangladesh

**Keywords:** ACE2, COVID‐19, delta variant, mutations, SARS‐CoV‐2, spike protein

## Abstract

**Introduction:**

Many of the global pandemics threaten human existence over the decades among which coronavirus disease (COVID‐19) is the newest exposure circulating worldwide. The RNA encoded severe acute respiratory syndrome coronavirus 2 (SARS‐CoV‐2) virus is referred as the pivotal agent of this deadly disease that induces respiratory tract infection by interacting host ACE2 receptor with its spike glycoprotein. Rapidly evolving nature of this virus modified into new variants helps in perpetrating immune escape and protection against host defense mechanism. Consequently, a new isolate, delta variant originated from India is spreading perilously at a higher infection rate.

**Methods:**

In this study, we focused to understand the conformational and functional significance of the missense mutations found in the spike glycoprotein of SARS‐CoV‐2 delta variant performing different computational analysis.

**Results:**

From physiochemical analysis, we found that the acidic isoelectric point of the virus elevated to basic pH level due to the mutations. The targeted mutations were also found to change the interactive bonding pattern and conformational stability analyzed by the molecular dynamic's simulation. The molecular docking study also revealed that L452R and T478K mutations found in the RBD domain of delta variant spike protein contributed to alter interaction with the host ACE2 receptor.

**Conclusions:**

Overall, this study provided insightful evidence to understand the morphological and attributive impact of the mutations on SARS‐CoV‐2 delta variant.

## Graphical abstract

COVID‐19 is a global pandemic that has been emerged as the most dangerous health threat of the recent decade. A newly discovered viral strain of Coronaviridae family named SARS‐CoV‐2 is the responsible infectious agent that causes COVID‐19. SARS‐CoV‐2 is a highly mutagenic virus by nature and recently a new variant of this virus named delta variant has been originated from India that spread drastically worldwide within few months. In this study, we analyzed the impact missense mutations found in the spike glycoprotein of delta variant.

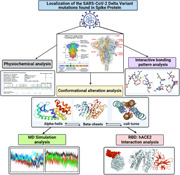

## 1 INTRODUCTION

The ongoing coronavirus disease (COVID‐19) pandemic caused by a novel virus from unknown origin, namely severe acute respiratory syndrome coronavirus 2 (SARS‐CoV‐2) is strikingly threatening the human health around the world. It is speculated that SARS‐CoV‐2 is a newly emerged zoonotic virus and the putative host for the transmission from animal to human is bat but there is no concrete scientific proof in this regard.^1^, ^2^, ^3^ Whereas, some scientists prefer to use the term “spill‐over” or “evolutionary jump” for transmission from animal to human which might be possible by random chance, repeated exposure or novel genetic changes.^2^ Pointing out the exact origin of SARS‐CoV‐2 is a matter of debate but in the meantime the total worldwide confirmed cases of COVID‐19 have exceeded more than 544 million with 6.3 million fatalities as of June 30, 2022. Structurally it is a novel single‐stranded RNA virus which is categorized in the Coronavirinae subfamily branching from the family *Coronaviridae* and the order Nidovirales. It was firstly mistaken as pneumonia with symptoms like fever, cough, difficulty in breathing, headache, sore throat, loss of taste or smell, nausea, and diarrhea.^4^, ^5^

Frequent mutations in the SARS‐CoV‐2 genome give rise to new variants even deadlier than previous one is emerging apparently on regular basis. So far, four variants of concerns (VOCs) namely B.1.1.7 (Alpha), B.1.351 (Beta), P.1 (Gamma), and B.1.617 (Delta) are dominating the world causing continuous trouble for the researchers and healthcare providers. According to the record of Pangolin server (https://cov-lineages.org),^6^ alpha variants was first traced in United Kingdom on 03‐09‐2020 then spread in 175 countries and total sequence count of this most infectious variant is 1,049,426.^6^ After first detection in South Africa on 01‐09‐2020, a total of 113 countries were affected by beta variant and total number of sequenced genomes after first detection is 29,473. On the other hand, gamma variant was emerged in Brazil 01‐10‐2020 caused devastating situation in 72 countries totaling 56,210 sequenced genomes. Finally, delta variant was first tracked out in India on 22‐09‐2020 caused unprecedented deaths in 152 countries for which total sequence count is 515,225. In the context of, total affected countries and sequence count, delta variant appeared as the most dominating variant of SARS‐CoV‐2 after alpha variant circulating worldwide.^6^

Entry of SARS‐CoV‐2 into the host cell is facilitated by spike glycoprotein (S protein) specifically by interaction and binding of S protein receptor‐binding domain (RBD) with the host cell human angiotensin converting enzyme 2 (hACE2) receptor.^7^, ^8^ In alpha variant, a total of 17 defining mutations were found and among these mutations 6 missense mutations specifically N501Y, A570D, P681H, T716I, S982A, and D1118H are in the S protein while in beta variant 6 missense mutations (D80A, D215G, K417N, A701V, N501Y, E484K) are in S protein were detected among total 9 mutations. Comparatively higher number of defining mutations (16) were mapped in gamma variant than other VOCs, where L18F, T20N, P26S, D138Y, R190S, K417T, E484K, N501Y, H655Y, and T1027I are in the S protein of SARS‐CoV‐2.^6^ Delta variant contains total 12 mutations in its genome and 9 missense mutations of interest in its S protein particularly T19R, T95I, G142D, and G158R in N‐terminal domain (NTD); L452R and T478K in RBD; D614G and P681R in hinge region of S1‐S2 subunit; D950N in the heptapeptide repeat sequence 1(HR1) of S2 subunit.^7^

In case of delta variant, distribution of mutations in S protein were resembling in appearance compared to the other variants. The NTD mutations G158R and T19R were mapped in the “supersite” of S protein and these altered residues suggested the reduced sensitivity of anti‐NTD neutralizing antibodies.^9^ This variant also harbors important L452R mutation in the RBD which impairs antibody neutralization.^10^ Furin cleavage site is significant for viral entry and presence of the P681R mutation in this region provokes the hypothesis that increment of S1–S2 cleavage at furin site is favored by P681 mutation resulting in enhanced infectivity of delta variant.^11^ Presence of the T478K mutation at the S protein‐hACE2 interaction interface is considered to elevate the surface electrostatic potential of S protein to even more positive value. Appearance of the D614G and D950N mutations in the trimer interface might have a role in the regulation of dynamics of S protein.^12^

Here we investigated nine of these nonsynonymous mutations in S protein of delta variant to analyze their biomolecular impact on the structure of wild‐type and mutant‐type. A wide range of analytical methods were performed in this research study such as physiochemical properties analysis, prediction of the changes in interactive bonding pattern, conformational alterations due to the targeted mutations, structural stability analysis, molecular docking and simulation study.

Thus, this computational study aimed to predict the impact of the missense mutations found in S protein of the SARS‐CoV‐2 delta variant on conformational stability of the protein and host‐virus interaction. The findings of this study hope to be very effective for understanding the reasons behind the effect of the concerning mutations on invading the host immune system and more transmissibility of this variant.

## 2 MATERIALS AND METHODS

### 2.1 Retrieval and processing of protein structure

The crystallographic conformation of SARS‐CoV‐2 spike protein trimer combined with P17 and FC05 Fabs cocktail (PDB ID: 7CWU) was retrieved from the protein data bank (https://www.rcsb.org/). After that, the trimeric structure of the S protein was extracted from the selected complex by using PyMol 2.4.0 version of Schrödinger platform.^13^ To understand the biomolecular interaction between SARS‐CoV‐2 RBD of the S protein and host ACE2 (hACE2) receptor, the crystallographic complex structure of RBD domain bound with hACE2 receptor (PDB ID: 6M0J) was also retrieved from the protein data bank. The targeted mutations were inserted according to the amino acid sequences and subjected to the domain‐specific mutated model development by using Swiss‐model platform.^14^ These preprocessed crystallographic wild and mutated structures are used in further analytical procedures of this study.

### 2.2 Comparative physiochemical properties analysis between the wild and mutated S protein

A protein's physiochemical characteristics aid in the understanding of biochemical and functional features of individual proteins—such characteristics take crucial part in determining the effect of certain mutations. Following this study, the physiochemical characteristics of the wild and mutant S protein of SARS‐CoV‐2 were compared using publically accessible Innovagen peptide analyzer (https://pepcalc.com/). This webserver evaluates the physiochemical characteristics of proteins based on their molecular weight, net charge at pH, isoelectric point, predicted solubility, extinction coefficient and hydrophilicity. The hydrophilic layout of the amino acid sequences is represented by using the hydropathy plot documented from Hopp and Woods model.^15^ The FORTRAN code‐mediated interactive HYDRO program is used in this model that stores the graphical peaks based on the hydrophilic profile of the amino acids. This optimized hydrophilic scale provides advantages to understand the relationship of protein sequence and interaction folds between the macromolecules from a surface‐exposed display.^15^

### 2.3 Analysis of the conformational alterations in spike glycoprotein

Characterization of the conformational alterations in secondary protein structures facilitates the comparative mutation analyses by determining the structural implications of mutations on the wild‐type and mutant protein. The Chou and Fasman Secondary Structure Prediction webserver analyses secondary structural regions including α‐helix, β‐sheets, and turns resulting from the correspondent amino acid sequence by implementing widely used Chou and Fasman algorithm with a combination of modern machine learning applications.^16^ According to the Chou‐Fasman method, the segments ≥6 residues with 〈P_α_〉 ≥1.03 as well as 〈P_α_〉 > 〈P_β_〉 (where P_α_ means helical propensities and P_β_ means corresponds to strand propensities) and under specific condition is predicted as helical structure. On the other hand, the segments ≥5 residues with 〈P_β_〉 ≥1.05 as well as 〈P_β_〉 > 〈P_α_〉 and under specific conditions, is predicted as β sheet. However, the turn structure is considered only if the probability of turn is greater than the probability of helix or sheet and a probability value based on particular amino acids in the turn oversteps the threshold parameter.^16^ Based on this formulation, the effect of nine targeted mutations on the conformation of spike glycoprotein was analyzed.

### 2.4 Prediction of the alteration in interactive bonding pattern due to the mutations

Alterations in morphological structure due to the missense mutations also leads to protein dysfunction by changing the interaction layout among the responding amino acids. Therefore, we analyzed the mutagenic impact of the targeted spike glycoprotein mutations of the SARS‐CoV‐2 delta variant by using PremPS server.^17^ This webserver is driven by the random forest regression scoring function and enriched with some other advanced methodological parameters. The *PSSM* and *ΔCS* parameter determine evolutionary conserved regions that function in folding of the protein. The *ΔOMH* is used to measure the fluctuation in hydrophobicity due to the mutation. The *SASA*_*sol*_ and *SASA*_*pro*_ indicates solvent accessible surface area (SASA) in the extended version of tripeptide and mutated protein region, respectively, which was determined by DSSP program.^17^ To identify whether the mutated residue is in the core region of the protein, the equation is used:

$$P_{x}=\frac{N_{x}}{N_{\mathrm{All}}}$$

Here *P*_*x*_ indicates probability index of the genetically altered amino acid (x), *N*_*x*_ represents the count of genetically altered amino acids and *N*_*All*_ represents the count of total amino acid residues. When the proportion of SASA for the mutated residue is less than 0.2, the amino acid is predicted to be buried in core region of the protein.^17^

### 2.5 Prediction of the differences in protein stability due to the targeted mutations

To evaluate the impact of all the targeted missense mutations of delta variant on the conformational stability of the SARS‐CoV‐2 S protein, we performed an analysis by using DynaMut webserver. It is a publicly accessible server that provides highly compendious package for protein stability prediction by combining the information from the DUET, Bio3D, and ENCoM. The prediction of protein stability formulates on basis of Gibbs free energy (ΔG) where the analysis is confirmed by blind tests and 10‐fold cross‐validation.^18^ The equation used to measure ΔG as follows:

ΔG=ΔH–TΔS;

Here, *ΔH* refers to difference in enthalpy, *T* is temperature, *ΔS* is difference in entropy.

The statistical metrics used to validate the analysis include Bivariate Correlation (r) and root mean squared error (RMSE).

$\text{Bivariate Correlation}\;\left(r\right),\rho_{X,Y}=\frac{\mathrm{cov}\left(X,Y\right)}{\sigma_{X}\sigma_{Y}}$

Here, cov(X,Y) appears for the covariance of *X* and *Y*, $\sigma_{X}$ and $\sigma_{y}\;$represents the standard deviation of the variable X and *Y*.

$$\mathrm{RMSE}=\sqrt{\frac{1}{n}\sum_{i=1}^{n}{\left(Y_{i}-{\hat{Y}}_{i}\right)}^{2}}$$

Here, n indicates total number of incidences and ${\left(Y_{i}-{\hat{Y}}_{i}\right)}^{2}$ determines the squared errors between actual observed values and the predictions.

### 2.6 Structural flexibility analysis by molecular dynamics (MD) simulation study

The MD simulation study of the protein and mutated constitutes was analyzed by YASARA dynamics assisted with the AMBER14 force field.^19^, ^20^ First of all, the targeted structures were polished, optimized and sequenced according to hydrogen bond network. The cubic simulation cell was created where the TIP3P solvation model was used with periodic boundary conditions.^21^ Then, simulation environments of the cells were settled with pH 7.4, 310 K, and 0.9% NaCl. After that, the simulated annealing methods (5000 cycles) were used to initial energy minimization administered by the steepest gradient algorithms.^22^ The time step of the simulation cell was settled at 2.0 fs. The Particle Mesh Ewalds methods were utilized to calculate the long‐range electrostatic interactions following a threshold radius of 8.0 Å.^23^, ^24^, ^25^ Utilizing the constant pressure and Berendsen thermostat, the simulation was conducted for 200 ns and after every 100 ps simulations trajectories were recorded. The recording simulation trajectories after constant intervals were used to measure the hydrogen bond, root mean square deviations, (RMSDs) radius of gyration and solvent accessible surface area.^26^, ^27^, ^28^, ^29^, ^30^, ^31^

### 2.7 Molecular docking study and analysis of interaction properties

We analyzed the interaction of host ACE2 receptor with the wild and mutated RBD by using HDOCK server. This is a comprehensive suit for template‐free docking study by implementing hybrid algorithm in which many of the experimental information including docking site localization, small‐angle X‐ray scattering scan can be observed. It provides top‐10 docked results on the basis of docking score and lowest RMSD.^32^ RMSD measures the interspace between the predicted and wild atoms of targeted protein by observing the similarity index of the conformations.^33^ The metrics used to calculate the RMSD as follows:

$$\mathrm{RMSD}=\sqrt{\frac{1}{N}\sum_{i=1}^{N}\delta_{i}^{2}}$$

Here, *N* indicates the count of atoms present in the ligand molecule and δ_*i*_ represents Euclidean distance between *i*th set of the respective atoms.

The interactive bonds between the wild RBD‐hACE2 and mutated RBD‐hACE2 were analyzed the using the Dimplot tool of LIGPLOT^+^ version 2.2.4. It represents the illustrative layout of the bonding pattern such as hydrogen bond, hydrophobic bonds between the two protein structures.^34^

## 3 RESULTS

### 3.1 Targeted mutations of delta variant contributed in changing the physiochemical properties of the spike glycoprotein

**Figure 1:**
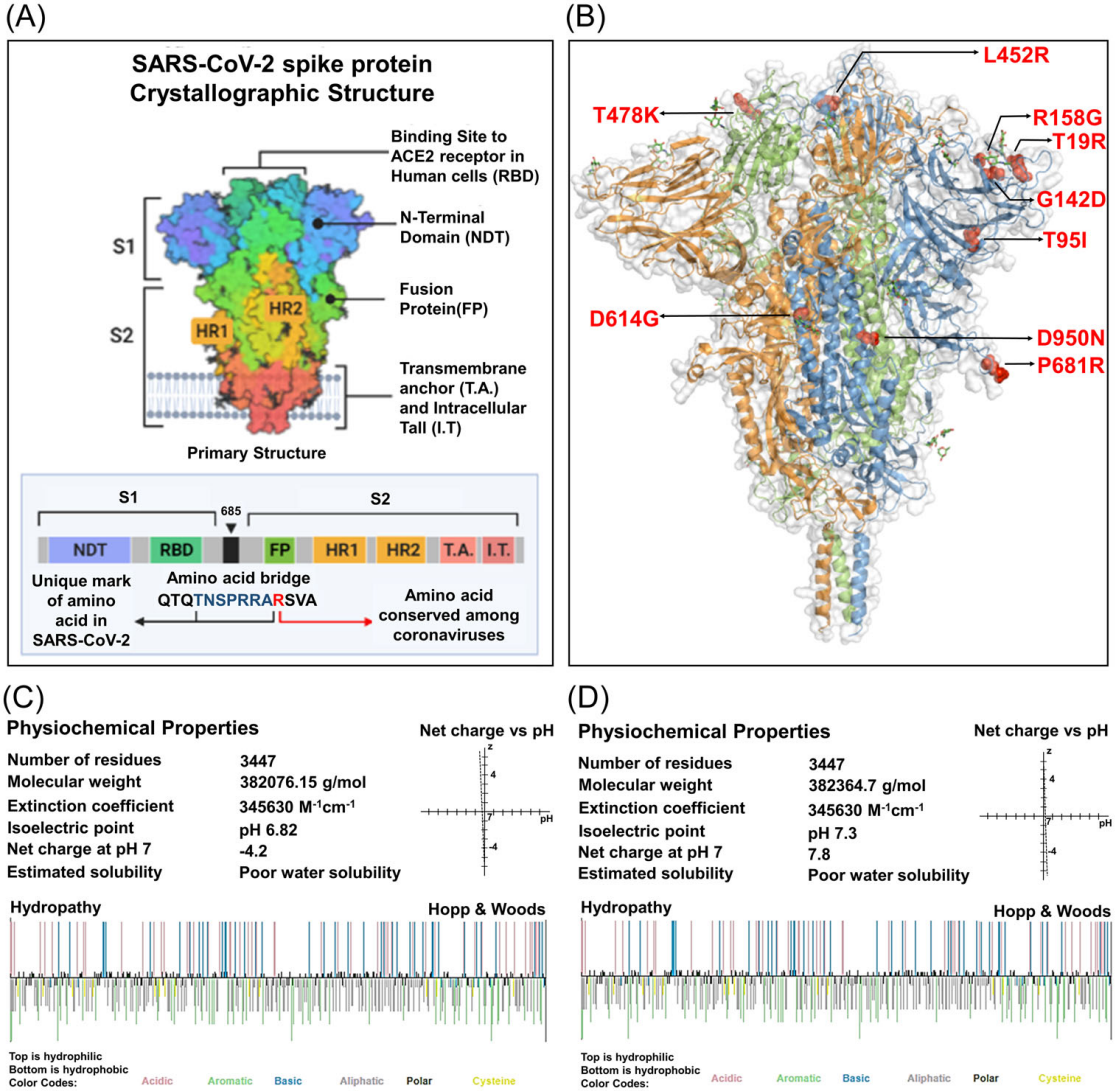
A comparative analysis to determine the impact of targeted mutations on the physiochemical properties of S protein. (A) Crystallographic conformation of the SARS‐CoV‐2 S protein (B) Localization of the targeted nine missense mutations found in the delta variant. The three amino acid chains of the trimeric S protein were encoded by three different colors, blue, green, and orange. The mutations were marked by red ball‐stick shape. (C, D) Physiochemical properties of both of the wild and mutated S protein. Top layer of the Hopp & Wood hydropathy plot remarked hydrophilic amino acids and the bottom layer indicated the hydrophobic amino acids. SARS‐CoV‐2, severe acute respiratory syndrome coronavirus 2

The 1273aa long spike (S) protein of SARS‐CoV‐2 composed of two subunits, namely S1 and S2. Most of the mutations of S protein were identified in different domains of S1 subunit. Specifically, T19R, T95I, G142D, and R158G mutations were detected in the N terminal domain, L452R and T478K mutations were found in the RBD domain, D614G and P681R mutations were identified in the hinge region between the S1 and S2 subunit. However, the D950N is the only missense mutation of delta variant detected in the heptapeptide repeat sequence 1(HR1) of S2 subunit (Figure 1A,B).

We exhibited a comparative physiochemical analysis between the native and mutated S protein to examine the changes in molecular weight, extinction coefficient value, isoelectric point, net charge at pH, and estimated solubility resulting from the targeted mutations of delta variant. Here, we found that the molecular weight of the S protein elevated from 382076.15 g/mol to 382364.7 g/mol as a result of these mutations. The isoelectric point was converted slightly acidic form (pH 6.82) to basic form (pH 7.3) due to the mutations. The wild S protein was negatively charged (−4.2) at neutral condition meanwhile the mutated S protein was found positively charged (7.8) at pH 7. The R158G, T95I, and D614G mutations resulted in an increase in hydrophobicity, whereas T19R, G142D, L452R, T478K, and P681R mutations resulted in a reduction and the hydrophobicity value of 950 residue remained unchanged (Table 1). The Hopp and Woods hydropathy plot depicted color‐coded categorization of amino acids and their hydrophobic/hydrophilic state in the wild and mutated form of proteins (Figure 1C,D).

**Table 1:** Comparative hydrophobicity of the wild and mutated residues of S protein represented in this table

		Hydrophobicity of the wild and mutated amino acids	
Mutations		Wild	Mutant
T19R		−0.7	−4.5
T95I		−0.7	4.5
G142D		−0.4	−3.5
R158G		−4.5	−0.4
L452R		3.8	−4.5
T478K		−0.7	−3.9
D614G		−3.5	−0.4
P681R		−1.6	−4.5
D950N		−3.5	−3.5

### 3.2 Conformational modification of the spike glycoprotein resulted from the targeted mutations

The secondary conformation of a protein originates on the basis of its amino acid sequence. In this analysis, we examined the impact of targeted nine mutations on amino acid sequence those may change the secondary conformation of the S protein.

**Figure 2:**
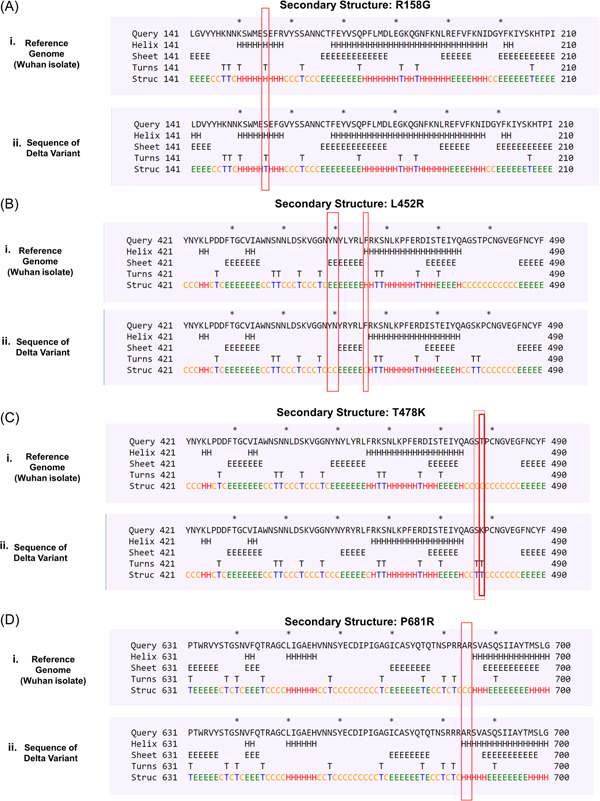
Conformational changes occurred as a result of the targeted mutations (A) R158G (B) L452R (C) T478K (D) P681R. The changes in structural conformation were marked in red box. Each of the structural type was encoded by one letter code: C—coil, E—β sheets, H—helix, T—turns

From the analysis we came to know that four mutations identified in the delta variant caused conformational change of the native S protein structure. Due to the R158G mutation, a previously existing helical structure at S155 residue was converted to a newly formed turn structure (Figure 2A). Two coiled‐coil conformations that existed in the wild type protein were substituted by two sheet structures located in the Y449 and N450 residues as a result of L452R mutation. A newly produced coiled‐coil structure was observed in the F456 region as well (Figure 2B. The T478 residue was leading to a coiled‐coil structure that was changed to a Turn type resulting from the T478K mutation. Apart from that, a coiled‐coil structure discovered in S477 has also been transformed to a Turn structure (Figure 2C). On the other hand, the P681R mutation caused the conversion of two coiled‐coil structure to helix structures at the adjacent A684 and R685 residues (Figure 2D).

Besides that, the consequences of rest of the mutations including T19R, G142D, D614G, and D950N on neighboring residues were found nonexistent as these mutations did not exhibit any kind of conformational changes (Figure S1).

### 3.3 Alterations in interactive bonding pattern of the spike protein due to the targeted missense mutations

**Figure 3:**
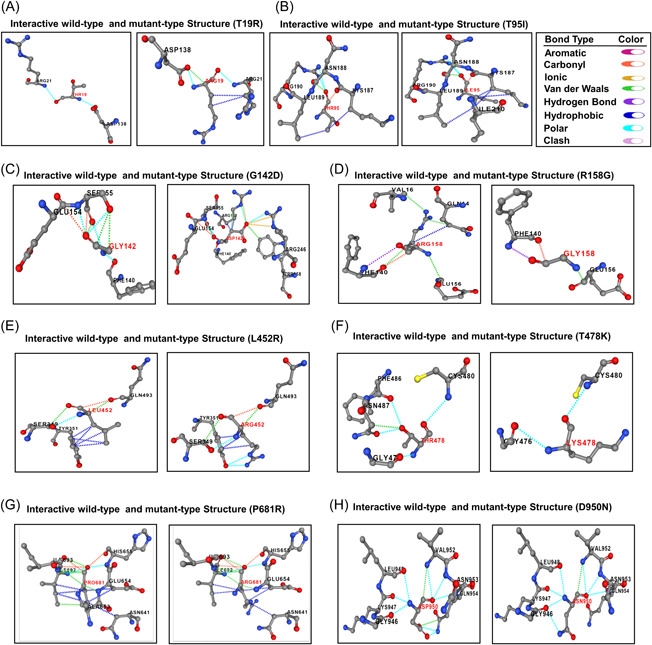
Alterations in interactive bonding pattern due to the mutations (A) T19R (B) T95I (C) G142D (D) R158G (E) L452R (F) T478K (G) P681R (H) D950N. Different bonds were encoded by specific color: hydrogen bond—violet, hydrophobic—deep blue, polar—sky blue, clash—pink, aromatic—dark pink, carbonyl—orange, ionic—yellow, Van daar Waals—green

Next, we analyzed the impact of targeted nine mutations on intramolecular bonding pattern to understand how they affect structural linkages of S protein. Considering the T19R mutation, we found that the natively generated polar between T19 and R21, T19 and D138 were remain unchanged, though the mutation leads to the formation of a new hydrophobic interaction between R19 and R21, and a new van der Waals bond between R19 and D138 (Figure 3A). Due to the T95I mutation, a new hydrophobic bond was formed between I95 and I210, with two other preexisting hydrophobic bonds between I95 and K187, I95, and L189. The polar bond between T95 and R190, carbonyl bond between T95 and K187 and van der Waals bond between T95 and N188 remained unchanged (Figure 3B). Following the G142D alteration, two new carbonyl bonds between D142 and R158, D142, and R246 were generated along with two other polar bonds between D142 and R158, D142, and R246. The previously existing interaction between G142 and S155 was rendered with additional two new van der Waals bond. A new hydrophobic bond was also formed between D142 and R158 on account of this G142D mutation (Figure 3C). Due to the R158G mutation, three van der Waals bond between R158 and Q14, R158 and V16, R158, and F140 were disrupted along with another hydrophobic bond between R158 and Q14 (Figure 3D). Resulting from the L452R mutation, the cross‐linked hydrophobic bond between L452 and Y351 was limited to pair‐based interaction. Two van der Waals bond between L452 and S349, L452 and Q493, and the only carbonyl bond between L452 and Q493 were also disrupted as a result of this mutation. However, a new polar bond between L452 and S349 was originated due to the mutation (Figure 3E). As a result of T478K mutation, the neutral threonine residue was replaced by a positively charged lysine residue and therefore, two polar bonds between T478 and F486, T478 and N487 were terminated along with a van der Waals between T478 and N487 (Figure 3F). A weak van der Waals bond between P681 and I692 was found to be eliminated because of the P681R mutation (Figure 3G). Similarly, one of the polar bonds between D950 and Q954, along with a van der Waals interaction were also knocked out due to the D950N mutation (Figure 3H). Any kind of noticeable changes in the interactive bonding pattern was not found for D614G mutations. However, this analysis provided insightful evidences about the effect of most of the targeted mutations in terms of interactive linkages among the amino acid residues of delta variant S protein.

### 3.4 The mutations of delta variant cause the modification of the S protein stability

Here, we calculated the difference of Gibbs free energy (**ΔΔG**) between the wild and mutant structure for each of the targeted mutations. The positive **ΔΔG** value indicates stabilization of the proteins whereas the negative **ΔΔG** value corresponds to the destabilization of the protein structure. Due to the T19R, T95I, and G142D mutations of NTD, we found the **ΔΔG** value of 0.120, 0.257, and 1.268 kcal mol^−1^, respectively, that results in stabilization of the mutated S protein structure. On the other hand, the **ΔΔG** value for R158G mutation was found −1.296 kcal mol^−1^ that indicates to the destabilization of the S protein. Following the RBD domain mutations, we found that L452R mutation had a negative **ΔΔG** value (−0.533 kcal mol^−1^) that caused destabilization of the protein. However, the T478K mutation was found to contribute in stabilizing the RBD domain as it possessed a positive **ΔΔG** (0.423 kcal mol^−1^) value. Considering the mutations of hinge region, we found that both of the hinge region mutations (D614G, P681R) mutation consisted of positive **ΔΔG** values (2.330, 0.236 kcal mol^−1^, respectively). The only considering mutation (D950N) of HR1 domain was found to stabilize the S protein as it expressed a positive **ΔΔG** value (0.064 kcal mol^−1^) (Table 2).

**Table 2:** Differences in Gibbs free energy due to missense mutations of SARS‐CoV‐2 S protein

			Gibbs free energy	
SL No.	Structural domain	Mutation type	Prediction outcome ΔΔG	Structural stability
1.	N‐terminal	T19R	0.120 kcal mol^−1^	Stabilizing
2.	N‐terminal	T95I	0.257 kcal mol^−1^	Stabilizing
3.	N‐terminal	G142D	1.268 kcal mol^−1^	Stabilizing
4.	N‐terminal	R158G	−1.296 kcal mol^−1^	Destabilizing
5.	RBD	L452R	−0.533 kcal mol^−1^	Destabilizing
6.	RBD	T478K	0.423 kcal mol^−1^	Stabilizing
7.	Hinge region	D614G	2.330 kcal mol^−1^	Stabilizing
8.	Hinge region	P681R	0.236 kcal mol^−1^	Stabilizing
9.	HR1 domain	D950N	0.064 kcal mol^−1^	Stabilizing

Abbreviations: RBD, receptor‐binding domain; SARS‐CoV‐2, severe acute respiratory syndrome coronavirus 2.

### 3.5 MD study indicates the impact of the mutations on structural flexibility of the delta variant

**Figure 4:**
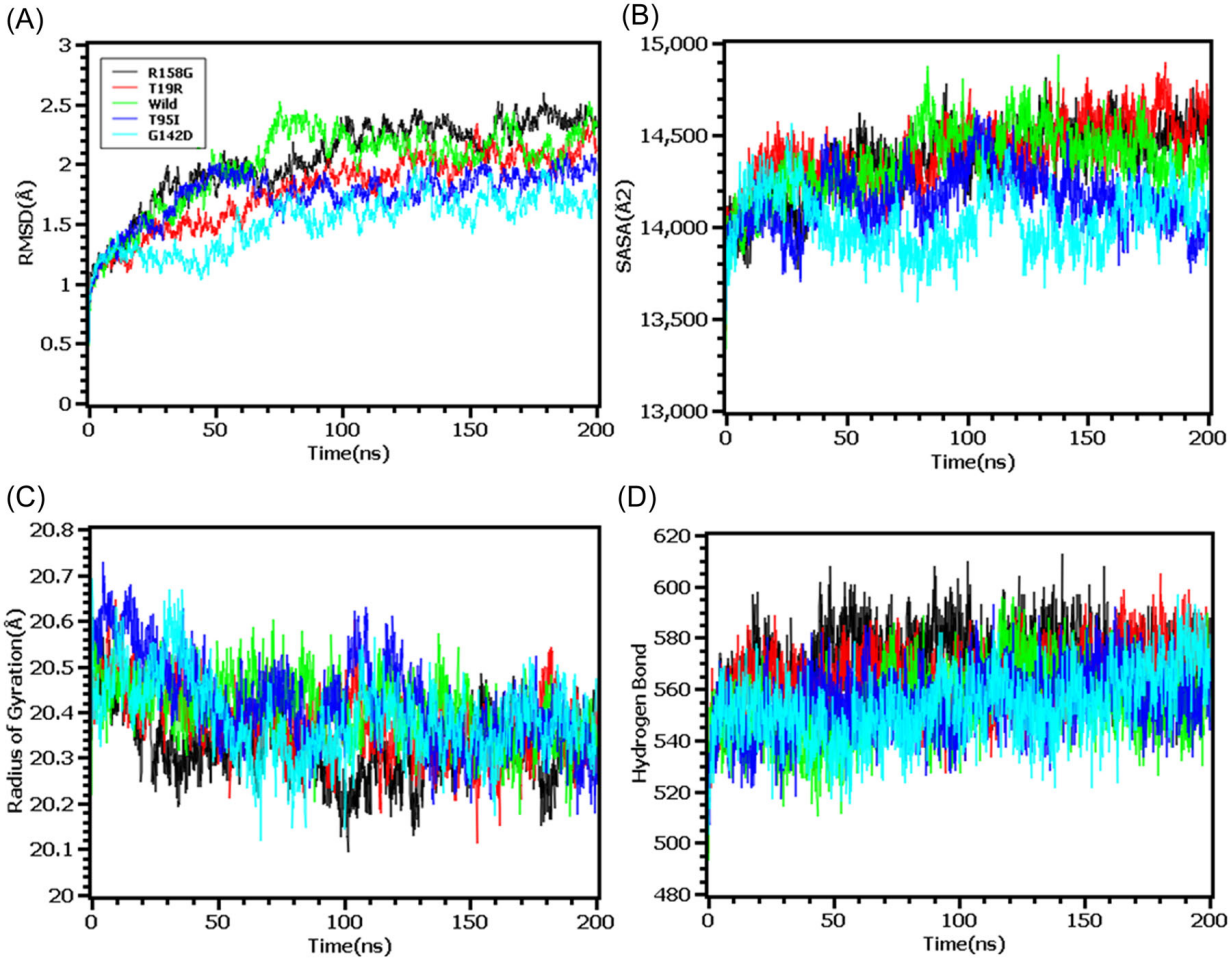
Molecular dynamics simulation study of the mutations of N‐terminal domain (T19R, T95I, G142D, and R158G) on different parameters (A) RMSD (B) SASA (C) Radius of Gyration (D) Hydrogen Bond. Every component of the system encoded by specific color, wild—green, T19R—red, T95I—deep blue, G142D—light blue and R158G—black. RMSD, root mean square deviation; SASA, solvent accessible surface area

The MD simulation study was conducted to understand the flexible nature of the variants across the simulation trajectories. First, MD of the wild type S protein and variants found in the N terminal domain were explored in simulation study where Figure 4A indicates that the RMSD of all the systems including wild type, R158G, T19R, T95I, G142D had initial rise in RMSD at the beginning. But the systems became stable after 100 ns time and maintained the integrity for the rest of the simulation periods. The SASA of T19R were higher than the other variants in simulations which indicates the flexible nature of this complexes (Figure 4B). All systems had shown lower deviation in rg profile and hydrogen bond patterning (Figure 4C,D).

**Figure 5:**
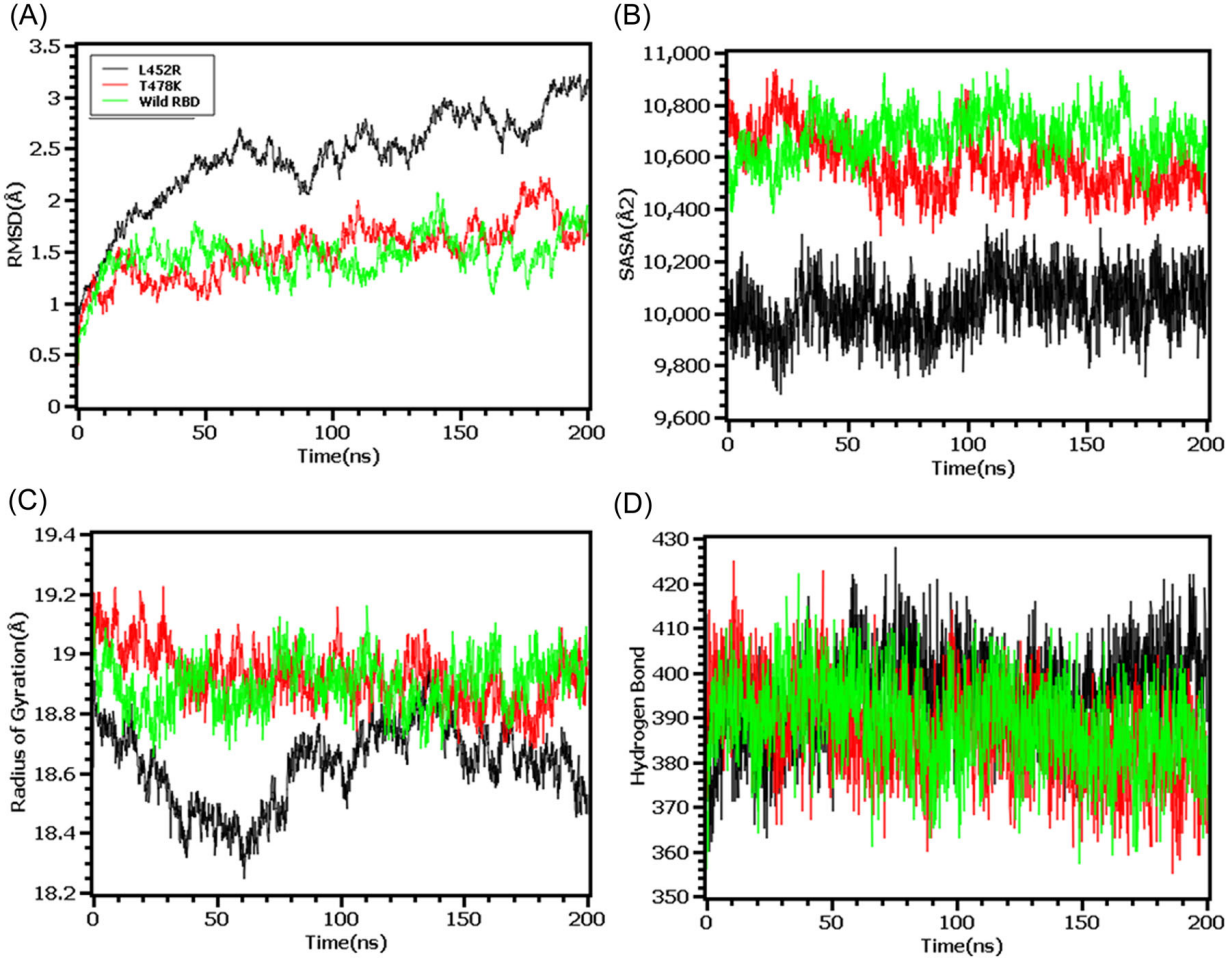
Molecular dynamics simulation study of the mutations of RBD (L452R and T478K) on different parameters (A) RMSD (B) SASA (C) Radius of Gyration (D) Hydrogen Bond. Every component of the system encoded by specific color, wild—green, L452R—black, and T478K—red. RBD, receptor‐binding domain; SASA, solvent accessible surface area

The RMSD profiling in RBD demonstrates that the wild types and T478K variants had similar RMSD and did not deviate much in the simulation trajectories. But the variants L452R had higher RMSD and deviations which correlates with the complexes less stable nature in the simulation systems (Figure 5A). The L452R variants had lowered its SASA than the wild types and T478K variants which indicates the condensed or truncated nature of this complexes (Figure 5B). Also, the Rg value of L452R was lower than the wild and other variants which indicates the flexible nature of this variant (Figure 5C).

**Figure 6:**
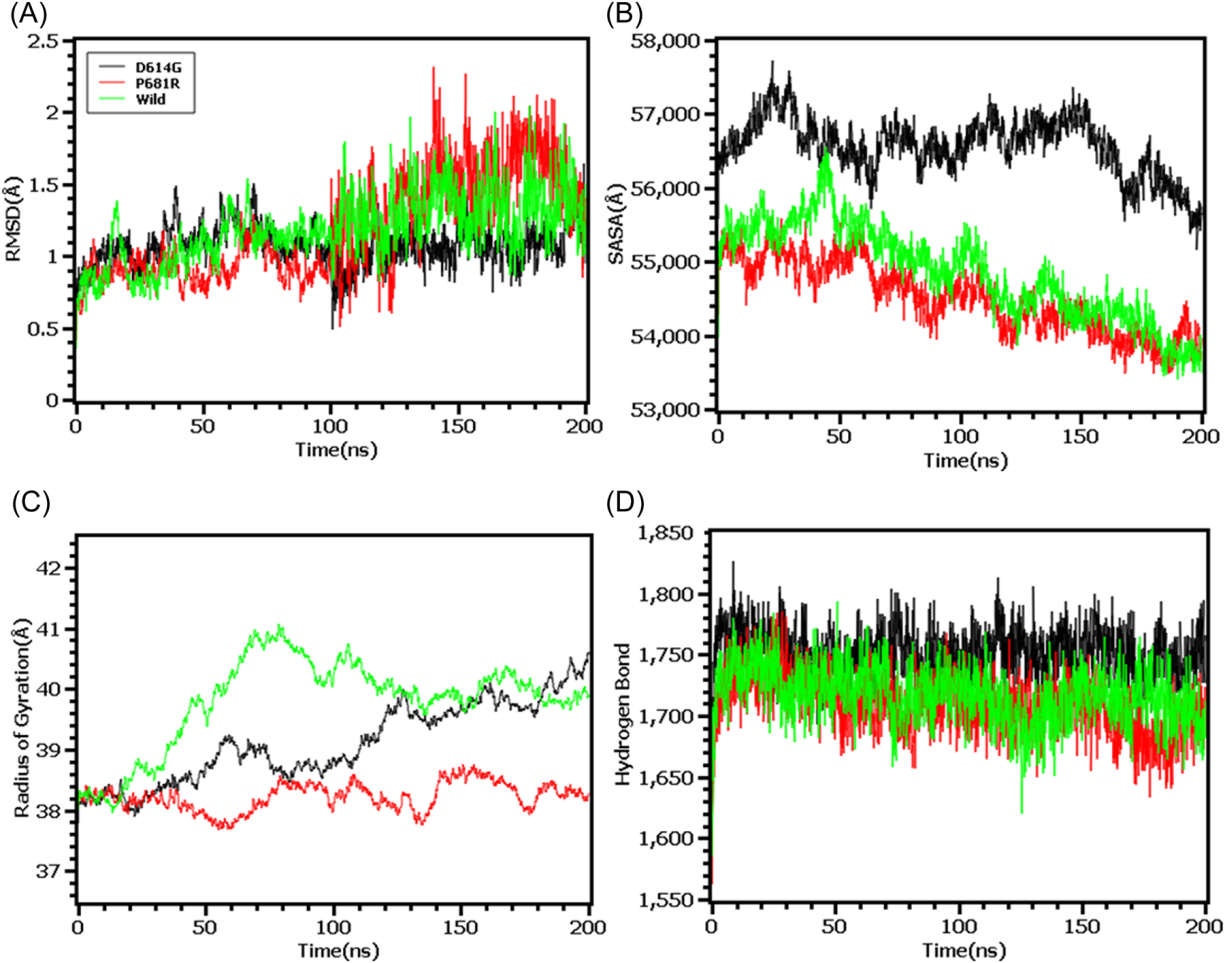
Molecular dynamics simulation study of the mutations of Hinge region (D614G and P681R) on different parameters (A) RMSD (B) SASA (C) Radius of Gyration (D) Hydrogen Bond. Every component of the system encoded by specific color, wild—green, D614G—black, and P681R—red. RMSD, root mean square deviation; SASA, solvent accessible surface area

The RMSD from the hinge regions was lower at the initial phase which might be responsible for the stable nature of the complexes. The D614G and wild types from the hinge regions had similar RMSD profile at the beginning to 50 ns times. The D614G complexes did not fluctuates much compared to the wild types and P681R variants which indicates more stable behavior of the D614G than the other complexes (Figure 6A). Therefore, the SASA of the hinge regions also explored to understand the changes in protein surface area where the higher SASA related with the expansion of the area and lower SASA related with the truncated nature of the complexes. The SASA was much higher for the D614G than the wild types and the P681R which indicates the protein expanded of its surface area upon mutations (Figure 6B). The P681R had lower Rg in the simulation's trajectories than the wild type and D614G which indicates the truncated nature of this variant compared to the wild type (Figure 6C). The hydrogen bond patterning of the three protein systems in hinge regions were stable and did not over fluctuate (Figure 6D).

**Figure 7:**
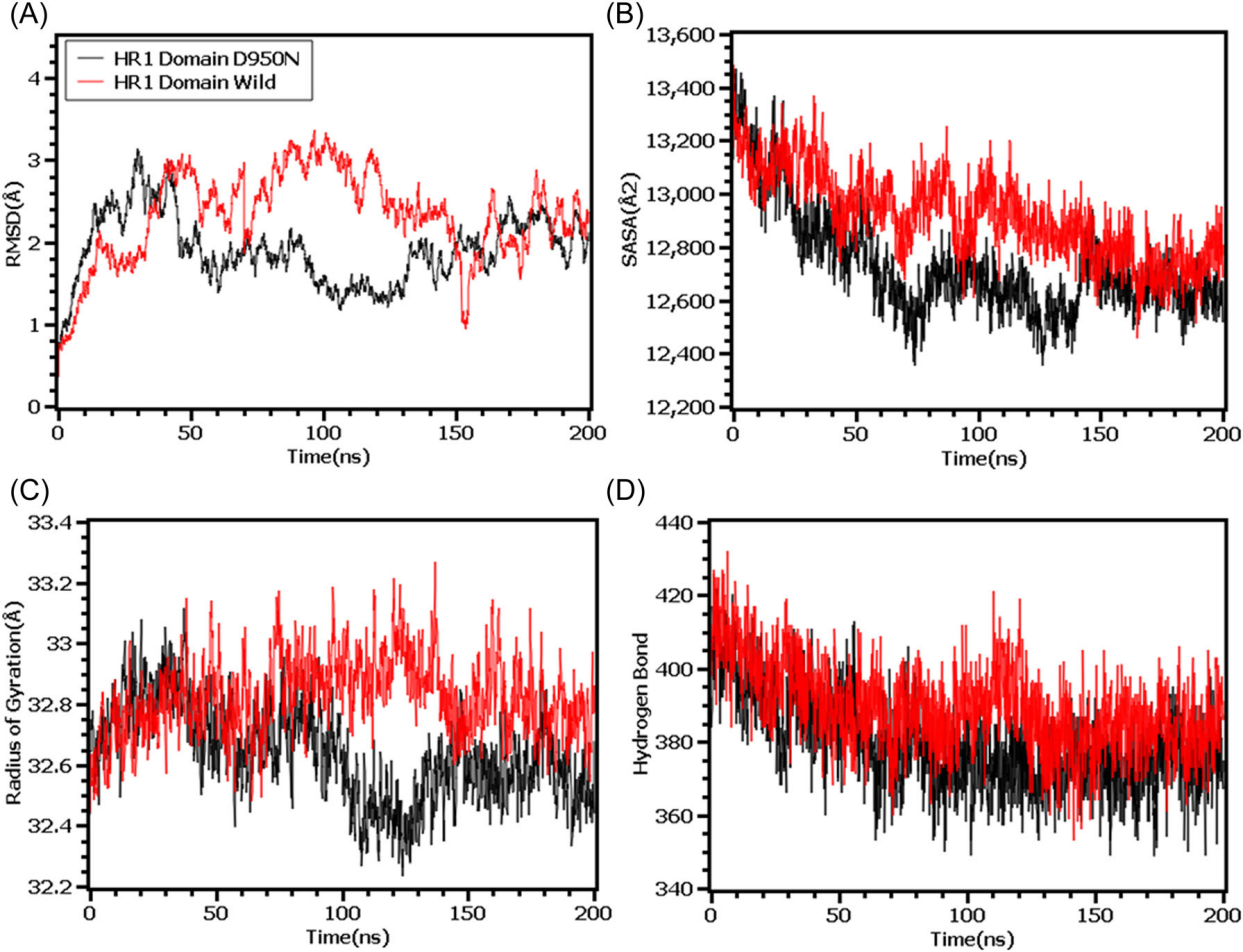
Molecular dynamics simulation study of the mutation of HR1 domain (D950N) on different parameters (A) RMSD (B) SASA (C) Radius of Gyration (D) Hydrogen Bond. Every component of the system encoded by specific color, wild—red and D950N—black. RMSD, root mean square deviation; SASA, solvent accessible surface area

Finally, the RMSD from the HR domain was also explored where D950N mutations was observed. The RMSD curve of wild type HR domain and D950N variant were initially upregulated. But the D950N variant had lower RMSD than the wild types in later parts of the simulations which defines comparative more stable nature of this complexes (Figure 7A). The SASA of D950N had slightly lower than the wild types which indicates the retention of the variant surface area (Figure 7B). The Rg value of D950N was also found quite lower than the HR wild type which correlates with the rigid nature of the complexes (Figure 7C). Therefore, the hydrogen bond pattern was stable for both systems in simulations (Figure 7D).

### 3.6 Mutations of delta variant alters the interaction between viral spike protein and ACE2

**Figure 8:**
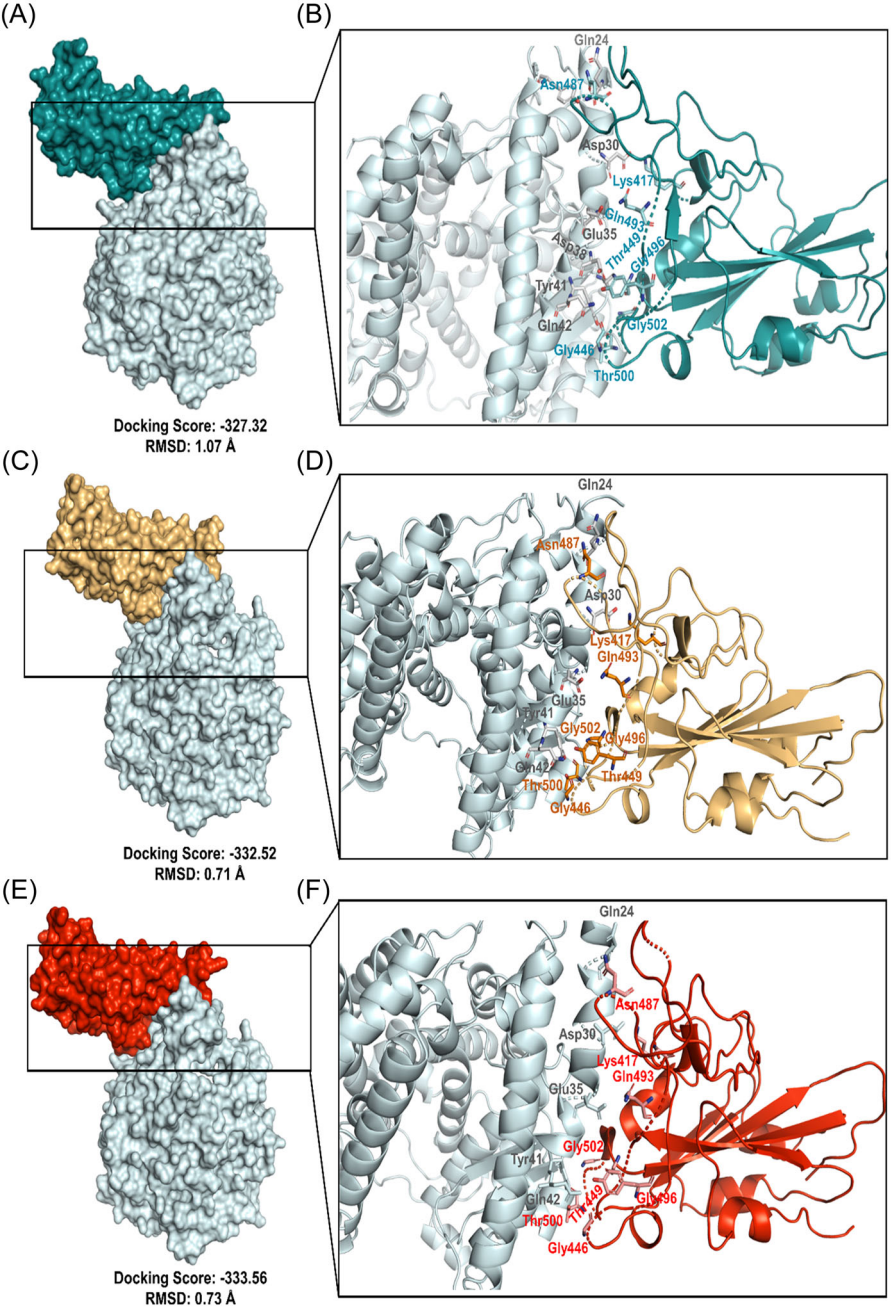
Molecular docking study to compare the interactive lineage between the wild RBD‐hACE2 and mutated RBD‐hACE2. (A, B) Interaction between wild RBD‐hACE2 (C, D) Interaction between L452R RBD‐hACE2 (E, F) Interaction between T478K RBD‐hACE2. hACE2, human angiotensin converting enzyme 2

RBD of the S protein interacts with host ACE2 (hACE2) receptor to get entry inside the host cell. In SARS‐CoV‐2 delta variant, two mutations (L452R and T478K) were detected in the RBD domain. Following this analysis, we elucidated the effect of these two mutations on RBD‐hACE2 interaction. First, we performed a docking analysis among wild RBD: hACE2, L452R RBD: hACE2 and T478K RBD: hACE2 where the interactive fitness was evaluated in terms of docking score and RMSD value. Here, we found comparatively better docking score for L452R RBD: hACE2 (−332.52), T478K RBD: hACE2 (−333.56) mutations than the wild RBD: hACE2 (−327.32). In terms of RMSD value, we also found that L452R RBD: hACE2 (0.71 Å) and T478K RBD: hACE2 (0.73 Å) exhibits lower RMSD score than the wild RBD: hACE2 (1.07 Å) interaction (Figure 8A–C).

**Figure 9:**
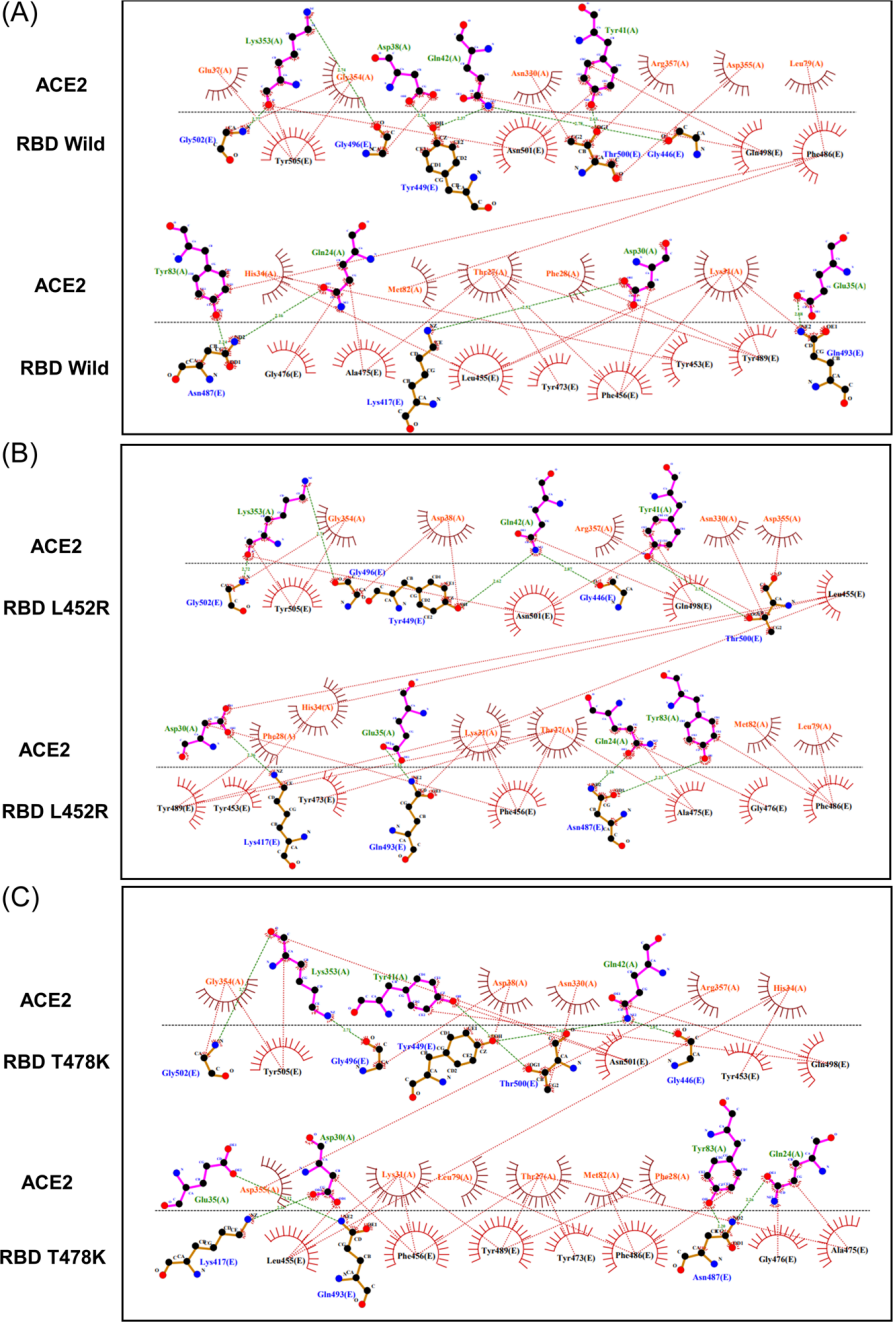
Comparison of the interactive bond formation found in wild RBD‐hACE2 and mutated RBD‐hACE2. (A) Interaction between wild RBD‐hACE2 (B) Interaction between L452R RBD‐hACE2 (C) Interaction between T478K RBD‐hACE2. H bonds were marked in green color and hydrophobic bonds were marked in red color. hACE2, human angiotensin converting enzyme 2; RBD, receptor‐binding domain

We extended this analysis to observe the alteration in bonding pattern of RBD: hACE2 interaction due to the two RBD domain mutations. Following this analysis, we compared the Hydrogen bonds (H bonds) and hydrophobic bonds of wild RBD: hACE2 with the L452R RBD: hACE2 and T478K RBD: hACE2 mutations by using the DIMPLOT tool of Ligplot v.2.2. Here, we identified 10 H bonds between the amino acid residues of wild RBD: hACE2 including K417: D30, G446: Q42, Y449: D38/Q42, N487: Q24/Y83, Q493: E35, T500: Y41, G502: K353 and G496: K353. Additionally, 24 hydrophobic bonds were also determined between wild RBD: hACE2 which are T453:H34, L455: D30/K31/H34, F456: T27/D30/K31, Y473: T27, A475: Q24/T27/, G476: Q24, F486: L79/M82/Y83, Y489: T27/F28/K31, Q498: Y41/Q42, N501: Y41/K353 and Y505: E37/K353/G354. But due to the L452R and T478K mutations, Y449: D38H bond and Y505:E37 hydrophobic bond were disrupted (Figure 9A–C). Overall, these two mutations of RBD were found to affect not only the binding affinity but also interrupted the bonding morphology of RBD: hACE2 interaction.

## 4 DISCUSSION

The B.1.617.1 variant of SARS‐CoV‐2 commonly referred as delta variant has currently become worrisome variant among all the variants circulating around the globe, responsible for recent upsurge in deaths and infections rate. Delta variant possesses nine signature mutations namely T19R, T95I, G142D, T148G, L452R, T478K, D614G, 681R and D950N NTD, RBD and the hinge region of S1–S2 subunit and HR1 domain of S protein, respectively. These mutations are plausible for increased infectivity, resistance to neutralizing antibodies and elevated transmissibility of delta variant. With this background, we investigated the biomolecular impact of these mutations to shed light on stability of delta variant S protein, its interaction with ACE2 receptor and understanding of immune evasion by delta variant.

Modification of an amino acid due to the mutations that occur in viruses is a natural phenomenon enabling them to adapt and survive in adverse physiological conditions by improving their fitness. RNA viruses including coronaviruses acquire mutations through three distinct mechanisms, and these mutations may be beneficial, deleterious, or neutral. The first mechanism is intrinsic, which implies errors in the proofreading system of RNA polymerase during viral replication facilitate the emergence of mutations. Secondly, recombination events between two lineages of viruses contribute to the rise of mutations. And, thirdly host RNA editing mechanism give rise to genomic diversity.^35^, ^36^ Surface protein mutations can drastically alter the viral infectivity, transmissibility, and their interactions with neutralizing antibodies.^37^ Diehl et al.^38^ and Urbanowicz et al.^39^ reported that increased infectivity and mortality of the Ebola virus are connected to the A28V mutations of the Ebola virus glycoprotein (GP). Tsetsarkin et al.^40^ reported that A226V mutation in the Envelope protein (E1 protein) of the Chikungunya virus resulted in increased transmissibility. In another study, Ning et al., demonstrated decreased reactivity of Avian influenza H7N9 to neutralizing antibodies as a result of A143V/R148K dual mutations of hemagglutinin protein.^41^

SARS‐CoV‐2 uses an intrinsic error‐prone RNA polymerase in its replication process and for that, it has a relatively higher mutation rate than DNA viruses. As a result, until May 2021 SARS‐CoV‐2 harbored an average of 10.24 ± 1.58 mutations in the S protein. Though most of them appeared to be non‐detrimental in nature, but there are still some mutations in the S protein that have significantly altered its infectivity and transmissibility.^42^ Rambaut A, et al.^43^ reported that the B.1.1.7 variant containing nonsynonymous spike protein mutations namely N501Y, A570D, D614G, P681H, T716I, S982A, D1118H found to be responsible for enhanced biding affinity of spike protein to hACE2 receptor, replication fitness and immune evasion. Xie et al.^44^ attested that the B.1.351 variant carrying E484K, K417N, and N501Y mutations in the spike protein reduced the reactivity of neutralizing antibodies. Shishir et al.^45^ analyzed the effects of spike protein mutations on the B.1.1.529 variant which is the most genetically diverse variant to date, and concluded with the findings that mutations in the spike protein of the omicron variant might be associated with the elevated transmission, risk of reinfection, decreasing vaccine effectiveness as well as hindering diagnosis.

Mutational changes of amino acids in a protein structure may results in misfolding of the protein, alterations of intermolecular interaction, shift of amino acids from buried to solvent exposed or solvent exposed to buried etc.^46^ From this perspective, we performed comparative physiochemical analysis of native and delta variant S protein, hence found changes in molecular weight, isoelectric point, overall charge and hydrophobicity. The molecular weight was higher in mutated version than native S protein whereas the iso‐electric point of wild protein was converted from acidic pH to basic condition due to the mutation. The overall charge was also shifted from negative to positive at neutral pH in mutated version of S protein. But, overall hydrophobicity of the mutated S protein reduced due to these mutations. The difference in physiochemical properties between native and delta variant S protein due to these signature mutations might be responsible for altering functional attributes.

Secondary structure of a protein is stabilized by hydrogen and hydrophobic interactions between residues and formation of more beta sheets in a protein provide higher chance of forming hydrophobic interactions.^47^ In the context of structural modifications out of nine four mutations particularly, R158G, L452R, T478K and P681R in the S protein of delta variant caused conformational transition from helical to turn, coiled‐coil to sheet, coiled‐coil to turn and coiled‐coil to helix. Structural modifications often result in alteration of the bonding pattern and by analyzing the bonding pattern of mutated S protein we came to know that, T19R, T95I, and G142D mutations introduced new polar, hydrophobic, carbonyl bond and van der Waals bond but interestingly did not alter the existing bonds between the respective residues. For G142D, R158G, L452R, T478K, P681R, and D950N mutations, existing polar, carbonyl, hydrophobic and van der Waals bonds between respective residues were knocked out, but L452R mutation introduced one polar bond. In our analysis, there was no significant changes in bonding pattern for D614G mutation. Therefore, it is evident from structural and intermolecular bonding pattern analysis that these altered secondary structure and bonding pattern may significantly enhance stability of delta variant S protein and increased affinity towards ACE2 receptor.

Structural changes of a protein structure namely alteration in cavity volume, packing density and solvent accessible surface area have ultimate effect on protein stability. The Gibbs free energy (**ΔΔG**) provides insightful information of structural stability of a protein by correlating with these parameters. In general, increase or decrease of protein stability is implies by positive or negative **ΔΔG** value, respectively.^18^, ^48^ In this study, out of nine analyzed mutations, positive **ΔΔG** value was observed for seven mutations indicating stabilization of mutated S protein, but R158G and L452R mutations of NTD and RBD of S protein respectively showed negative ΔΔG value causing destabilization of mutated S protein.

To observe natural phenomenon that is quite impossible to investigate experimentally, MD simulation is best fit for this purpose and successfully employed for predicting stability of a protein in a biological condition and from drug designing to screening.^49^ We carried out domain specific MD simulations to observe differences in flexibility between wild type and mutated S protein. RMSD is the measure of average deviation of a given atom between two proteins and lower RMSD refers to better stability.^49^, ^50^ On the other hand, Radius of gyration (Rg) depicts the tightness of a protein structure where higher Rg value depicts more stable structure.^51^ Besides, SASA illustrates the surface area of a molecule accessible to solvents where increased value of SASA defines the changes in protein surface volume of a protein.^52^ In terms of RMSD value, the mutations in the NTD, RBD, hinge region and HR domain of S protein were accounted for overall lower RMSD value compared to the native S protein with some fluctuations throughout the simulation period. Also, the SASA value of some mutations were comparatively lower than wild type but most of the mutations have higher SASA value than native S protein indicating expansion of the surface area. All of the mutations had stable Rg value across the simulation's trajectories and hydrogen bond than the wild type S protein throughout the simulation period which evidently indicate the stability of the S protein of delta variant over native protein.

Protein–protein interactions between viral protein and receptor of host cell mediates proper interaction and subsequent entry of a virus into the cell, hence alterations of pivotal residues in the binding site of a viral protein could increase or decrease binding efficacy.^53^, ^54^ To elucidate the effect of L452R and T478K RBD mutations on RBD: hACE2 binding and bonding pattern, we carried out molecular docking studies and compared the results with wild type RBD results. Interestingly, docking score for these two mutations were higher than the wild type RBD: hACE2 while RMSD value of docked complexes (L452R RBD: hACE2 and T478K RBD: hACE2) were lower than wild type which evidently signifies strong and stable binding of mutated RBD with hACE2. By observing bonding interaction of mutated RBD: hACE2 after molecular docking, it is found that, number of hydrogen and hydrophobic bonds was slightly reduced compared to the wild RBD: hACE2 due to the abolishment of Y449:D38H and Y505:E37 hydrogen and hydrophobic bonds respectively. However, the influence of missing interactions on stability of RBD: hACE2 complex of delta variant S protein is a matter of further investigation. In addition, Cherian et al., demonstrated that, enhanced stabilization of RBD:hACE2 is attributed to L452R and T478K mutations and L452R contributed to the 3.5 fold increase in the infectivity of delta variant^55^ which aligns with our findings.

Baral et al.^51^ reported that resistance to neutralizing antibodies and evasion of immune response by delta variant is attributed by RBD mutations (L452R and T478K) of the S protein because these mutations provided altered receptor binding interface than wild type S protein. Tada et al.^56^ depicts that L452R mutation contributed to the reduction in neutralizing potential of 14 out of 35 RBD specific monoclonal antibodies (mAbs) and in another study conducted by McCallum et al.^57^ showed that L452R mutations promotes viral replication hence infectivity of the virus by facilitating immune evasion from cellular immunity provided by the HLA‐24 (Human leukocyte antigen 24). Moreover, Yadav et al.^58^ investigated the neutralizing capacity of Covaxin and Covisheild vaccines and found almost twofold reduction in neutralization for Covaxin and even more reduction for Covishield against delta variant.^59^, ^60^, ^61^

To sum up, the spike protein of SARS‐CoV‐2 is crucial for viral infectivity and transmissibility in the host, therefore, any mutations would have definitive consequences on virulence as well as therapeutic efficacy. Hence, currently all the available vaccines, drugs, or neutralizing antibodies as protective measures are primarily focused on spike protein, it is pivotal to monitor the mutations of spike protein in the circulating variants. The findings of our study will aid the researchers to evaluate the efficacy of the approved vaccines administered around the globe and monitoring the ability of immune evasion of the mutated delta variant. More importantly, the findings will be helpful in the process of implementing targeted control measures against delta variant, and to tailor variant‐specific vaccines, therapeutics, and diagnostics measures.

## 5 CONCLUSIONS

To date, researchers and healthcare providers are trying relentlessly to sustain the infection by administering vaccine to people around the world and developing drugs for the treatment of infected individuals. But, frequent mutations in the SARS‐CoV‐2 genome can be advantageous for the virus resulting in elevation of transmission, infection and may limit the efficacy of available vaccines as well as causing potential threat to drug development process. To shed light on this matter, we employed several bioinformatics tools targeting nine signature mutations of delta variant S protein to find out the structural and conformational changes. In conclusion, our study points out a relation between mutations in different domains of the delta variant S protein and explores biological reasons behind the elevated pathogenicity, infectivity and immune escape of delta variant which might be useful for design and manufacture drugs and vaccines specially for delta variant.

## AUTHOR CONTRIBUTIONS

**Tousif Bin Mahmood**: conceptualization, methodology, formal analysis, writing—original draft. **Mohammad Imran Hossan**: writing—original draft, writing—review and editing. Shafi Mahmud: Formal analysis, Writing—original draft. **Mst. Sharmin Sultana Shimu**: formal analysis, writing—review and editing. **Md. Jahidul Alam**: formal analysis, writing—original draft. **Md. Mahfuzur Rahman Bhuyan**: formal analysis, writing—original draft. **Talha Bin Emran**: supervision.

## CONFLICT OF INTEREST

The authors declare no conflict of interest.

## Supporting information

Supporting information.
