# Supplementary material for: Missense mutations in spike protein of SARS‐CoV‐2 delta variant contribute to the alteration in viral structure and interaction with hACE2 receptor
Source: Immun Inflamm Dis. 2022 Aug 17;10(9):e683. doi: 10.1002/iid3.683 (PMC9382871; doi:10.1002/iid3.683)
Supplement: Supplementary file 1 — Supporting information. [file IID3-10-0-s001.docx]

**Supplementary Materials**


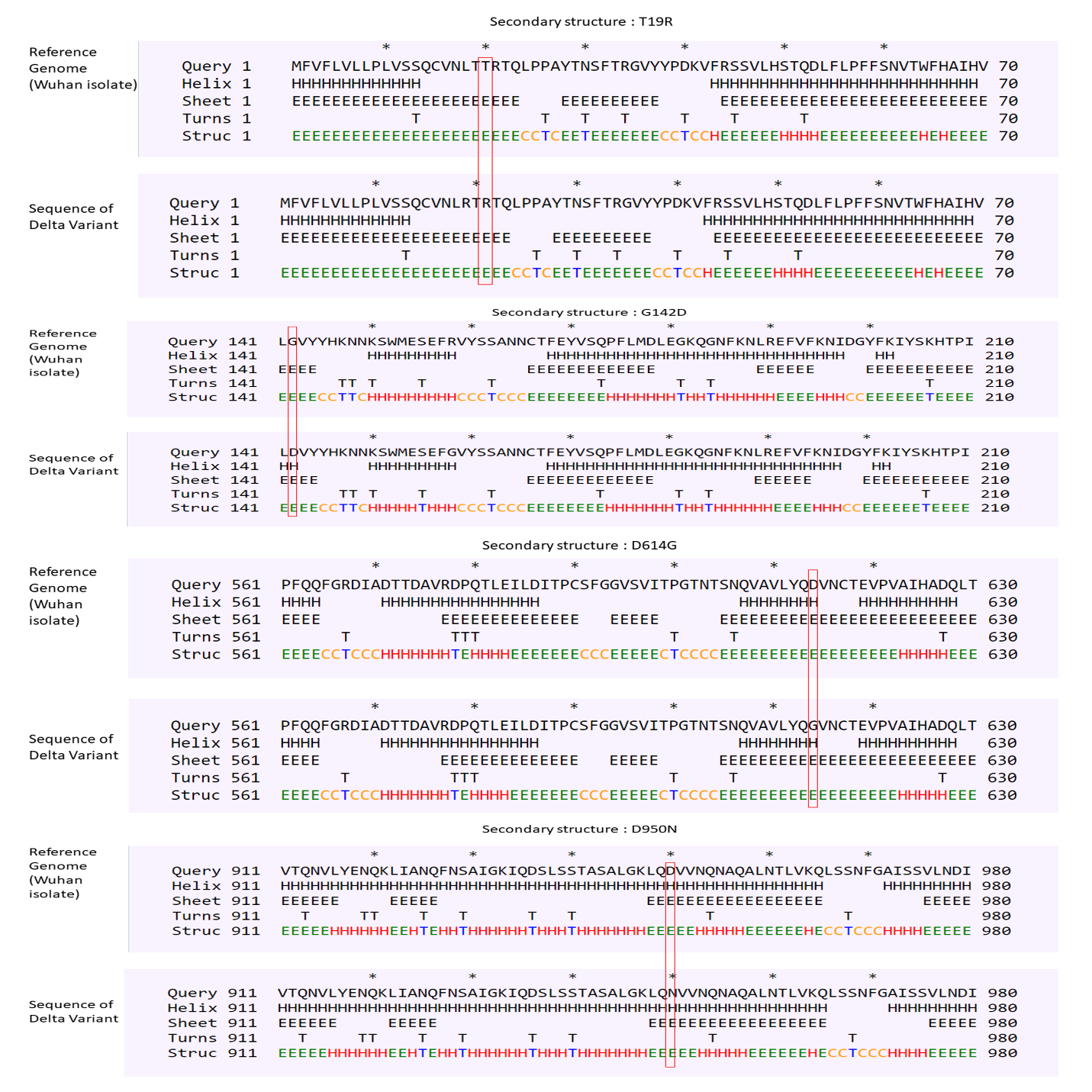


**Supplementary Figure 1.** Conformational analysis targeting the spike protein mutations **(A)** T19R **(B)** G142D **(C)** D614G **(D)** D950N. The mutated regions were marked in red box. Each of the structural type was encoded by one letter code: C – Coil, E – β Sheets, H – Helix, T – Turns.
